# Regional anesthesia in bariatric surgery

**DOI:** 10.1097/ACO.0000000000001506

**Published:** 2025-04-29

**Authors:** Alessandro De Cassai, Serkan Tulgar, Michele Carron, Paolo Navalesi

**Affiliations:** a Department of Medicine (DIMED), University of Padua, Padua, Italy; b Institute of Anaesthesia and Intensive Care, Padua University Hospital, Padua, Italy; c Department of Anesthesiology and Reanimation, Samsun Training and Research Hospital, Samsun University Faculty of Medicine, Samsun, Turkey

**Keywords:** bariatric surgery, multimodal analgesia, obesity, opioid reduction, regional anesthesia

## Abstract

**Purpose of review:**

Obesity presents significant perioperative challenges, particularly in bariatric surgery, where optimizing pain management while minimizing opioid use is crucial. Recent advancements in regional anesthesia (RA) techniques offer potential benefits in enhancing perioperative outcomes for this high-risk population.

**Recent findings:**

Current evidence supports the use of RA techniques such as transversus abdominis plane (TAP) block, quadratus lumborum (QL) block, erector spinae plane (ESP) block, and intraperitoneal instillation of local anesthetics in reducing postoperative pain and opioid consumption. While TAP and ESP blocks improve postoperative analgesia, the QL block offers longer-lasting pain relief. Intraperitoneal local anesthetic administration has shown potential in decreasing opioid use and improving respiratory recovery. Additionally, port-site infiltration remains a simple yet effective alternative. However, anatomical challenges in obese patients necessitate optimized ultrasound guidance for successful block placement.

**Summary:**

RA is a key component of multimodal analgesia in bariatric surgery, contributing to reduced opioid-related complications and improved recovery. Despite promising findings, further high-quality randomized controlled trials are needed to refine technique selection and enhance clinical outcomes in this patient population.

**KEY POINTS**

- Bariatric surgery presents unique challenges, including altered anatomy, increased perioperative risks, and the need for tailored anesthesia techniques to optimize patient safety and recovery.
- Regional anesthesia (RA) techniques, including transversus abdominis plane, quadratus lumborum, and erector spinae plane blocks, play a crucial role in multimodal analgesia for bariatric surgery, reducing postoperative pain and opioid consumption.
- Intraperitoneal local anesthetic instillation shows potential benefits in pain control and opioid reduction, though current guidelines do not yet widely endorse it.
- Port-site infiltration remains a simple and effective alternative to fascial plane blocks, with comparable analgesic outcomes in bariatric patients.
- Ultrasound guidance is essential for optimizing RA in obese patients, requiring specific adjustments to enhance visualization and success rates.

## INTRODUCTION

Obesity, defined by a BMI higher than 30 kg/m^2^ [1], represents one of the most significant global health challenges, with its prevalence steadily increasing over recent decades [2^■^]. This condition substantially elevates perioperative risks and postoperative complications, such as respiratory failure and thromboembolic events [3,4]. Moreover, patients with obesity often present with comorbid conditions such as obstructive sleep apnea syndrome (OSAS) [5] and obesity hypoventilation syndrome both related to physiological alterations in the obese population [6–8]. Which significantly increases the risk of postoperative complications such as desaturation, respiratory failure, and cardiovascular events [3,9]. Additionally, patients with obesity are also at an increased risk of postoperative nausea and vomiting (PONV) that is exacerbated by the concurrent use of opioids during general anesthesia [3,10] with an incidence ranging from 60 to 90% [10]. PONV is a prevalent issue among patients undergoing bariatric surgery, with an incidence rate ranging from 60 to 90% [10], the percentage remains high (20%) even when a triple prophylaxis approach is adopted [11].

National and international guidelines [12,13] recommend minimizing opioid use in obese surgical patients to reduce the risk of opioid-induced airway obstruction and respiratory depression [14], particularly in those with OSAS and respiratory disease [15]. Moreover, multimodal analgesia is endorsed to optimize pain control, reduce opioid consumption [16^■■^], and promote early mobilization, which is linked to fewer postoperative complications and shorter hospital stays [12,13]. While intravenous multimodal analgesia remains a cornerstone of perioperative care for obese patients, combining it with regional anesthesia (RA) offers a more effective approach, especially in bariatric surgery [17]. RA has shown significant benefits in reducing opioid consumption, postoperative pain scores, and the need for rescue analgesics in patients with obesity undergoing surgery compared to general anesthesia alone [18^■■^].

However, the implementation of RA in patients with obesity presents unique challenges, including anatomical variations, increased adipose tissue, and altered pharmacokinetics. These factors complicate the success of certain regional techniques and increase the risk of procedural failure [5,18^■■^]. This review aims to explore the latest advancements in RA techniques, evaluating their benefits, limitations, and practical applications in enhancing perioperative outcomes for patients undergoing bariatric surgery.

## INTRAPERITONEAL INSTILLATION OF LOCAL ANESTHETIC

Intraperitoneal instillation (IPLA) involves spraying local anesthetics (e.g. ropivacaine, bupivacaine, or lidocaine) into the peritoneal cavity, occasionally with adjuncts like dexmedetomidine or tramadol to enhance analgesic effects [19].

Recommendations for IPLA vary depending on the surgical context, with some guidelines questioning its benefits [19,20]. A 2024 meta-analysis of 24 randomized controlled trials (RCTs) (1705 patients) undergoing laparoscopic digestive surgery provided evidence supporting IPLA’s efficacy. Ropivacaine (0.2–0.75%, 20–50 ml) reduced pain scores within 24 h and 24-h morphine use by 21.9 mg compared to saline placebo [21]. Additional benefits included decreased postoperative shoulder pain, reduced PONV, and shorter hospital stays [21].

In patients with obesity undergoing laparoscopic surgery, current guidelines do not specifically recommend IPLA [22]. However, recent studies highlight its potential utility in patients undergoing laparoscopic bariatric surgeries [23–25]. Using bupivacaine (0.25%, 30 ml) compared to saline placebo significantly reduced pain scores during the first 6 h postsurgery. Morphine requirements via patient-controlled analgesia decreased by approximately 15%, with fewer additional analgesic needs reported [23].

Using ropivacaine (0.375%, 40 ml) compared to saline placebo significantly reduced pain scores within the first 24 h [24]. Pain at rest decreased by 72% [adjusted odds ratio (aOR) 0.28], while pain during movement decreased by 75% (aOR 0.25). No significant differences were observed in postoperative length of stay, total opioid use, antiemetic needs, morbidity, or mortality [24]. Additionally, intraperitoneal ropivacaine (40 ml of 0.5%) compared to placebo significantly improved respiratory recovery within the first 24 h, as measured by incentive spirometry. A reduced need for additional postoperative analgesia was also observed [25].

While current guidelines caution against recommending IPLA for bariatric surgery [22], recent evidence challenges this position. By reducing postoperative pain and opioid consumption and improving respiratory recovery, IPLA aligns with the principles of enhanced recovery after bariatric surgery. Further high-quality randomized controlled trials are required to validate its safety and efficacy in this population.

## TRANSVERSUS ABDOMINIS PLANE BLOCK

The transversus abdominis plane (TAP) block was introduced by Rafi in 2001 as a landmark-based, single-pop technique [26]. Its relatively short learning curve [27], even for nonanesthesiologist practitioners [28], has facilitated its integration into multimodal analgesia strategies [29].

The use of this block has yielded mixed results since the first randomized controlled trials in patients undergoing bariatric surgeries. While some studies reported reduced opioid requirements, improved pain scores, decreased sedation, earlier ambulation, and greater patient satisfaction [30], others found no significant differences [31]. A meta-analysis of 11 RCTs involving 789 patients undergoing laparoscopic bariatric surgery demonstrated that the ultrasound-guided TAP block, as part of multimodal anesthesia, significantly improved postoperative pain control and reduced 24-h morphine consumption by 32 mg, achieving an 80% reduction in the need for additional opioid doses [29].

It is important to highlight that this meta-analysis did not differentiate between the TAP block and the subcostal TAP block, instead combining both techniques into a single analysis. While both blocks target the same fascial plane, their areas of analgesia differ slightly. The TAP block, typically performed at the mid-axillary line between the iliac crest and the costal margin, primarily targets the lower thoracic and upper lumbar nerves, providing analgesia for the lower abdominal wall. In contrast, the subcostal TAP block is administered along the subcostal margin, closer to the xiphoid process, and targets more cephalad nerve branches, offering analgesia for the upper abdominal wall. Given these differences, it is reasonable to question whether one technique might be preferable to the other. However, in a recent network meta-analysis we conducted, TAP block demonstrated only a slight advantage over the subcostal TAP block in terms of both opioid consumption and postoperative pain [18^■■^].

## ERECTOR SPINAE PLANE BLOCK

The erector spinae plane (ESP) block, first described by Forero *et al*. [32], is a relatively recent addition to the family of fascial plane blocks. Although some controversies remain, its primary mechanism of action is believed to involve a paravertebral ‘by-proxy’ effect [33], however, variability in injectate spread raises concerns about its consistency across surgical contexts [34].

In bariatric surgery, the ESP block has garnered attention for its ability to address both somatic and visceral pain—key considerations in laparoscopic bariatric procedures. Early case series highlighted its potential [35], while subsequent RCTs confirmed its effectiveness in pain relief and as an intraoperative and postoperative opioid-sparing strategy [36–38]. ESP block with bupivacaine (0.5%, 20 ml) and lidocaine (0.2%, 5 ml) significantly reduced pain scores within 24 h, with no need for additional analgesia in the ESP group [37]. Using bupivacaine (0.25%, 20 ml), the ESP block significantly reduced pain scores within the first 8 h compared to control (normal saline, 20 ml). It also prolonged the time to first rescue analgesic and achieved an 8 mg reduction in 24-h morphine equivalent consumption with no significant differences in PONV or postoperative complications reported [36].

The ESP block’s effect on postoperative respiratory function remains uncertain [38]. Although patients with obesity showed significant baseline decreases in respiratory function [36], the ESP block did not improve spirometric values, arterial blood gas parameters, or respiratory recovery compared to control groups [36–38]. In bariatric patients, the ESP block showed slight statistical advantages over the TAP block in pain scores and rescue analgesia delays, but the clinical significance was limited [39].

The ESP block’s superficial location and favorable safety profile further enhance its appeal, particularly for patients on antithrombotic therapy. Unlike deeper blocks such as paravertebral or epidural techniques, the ESP block is associated with minimal risk of complications, such as pneumothorax or systemic toxicity [40,41].

The ESP block offers a safe and effective option for multimodal analgesia in bariatric surgery, reducing opioid consumption and providing satisfactory pain control. However, its inconsistent effects on respiratory recovery and marginal advantages over TAP block highlight the need for further high-quality RCTs to better define its role in this population.

## QUADRATUS LUMBORUM BLOCK

The quadratus lumborum (QL) block was first described nearly two decades ago as a modification of the TAP block [42]. Since then, several variations (QL1, QL2, and QL3) have been developed. Despite differences in nomenclature, the core principle remains the same—injecting local anesthetic around the QL muscle (laterally, posteriorly, or anteriorly). This technique aims to direct the anesthetic along the thoracolumbar and endothoracic fascia, ultimately reaching the paravertebral space. In bariatric surgery, the QL block has gained attention for its ability to reduce intraoperative and postoperative opioid consumption. When performed with bupivacaine [0.25%, 0.2 ml/kg lean body weight (LBW)], the QL block significantly reduced pain scores at rest and with movement, decreased intraoperative and postoperative opioid requirements, prolonged the time to first rescue analgesic, and lowered the incidence of nausea and vomiting compared to control (normal saline, 0.2 ml/kg LBW). It also enabled faster ambulation, with no significant differences in shoulder pain or other opioid-related side effects [43]. Compared to the ESP block, the QL block lasted longer but took more time to administer [44]. Additionally, the QL block demonstrated potential cognitive recovery benefits linked to reduced systemic inflammation, particularly lower interleukin-6 levels [45].

The QL block offers a promising option for multimodal analgesia in bariatric surgery, reducing opioid use and postoperative pain, with unique advantages such as longer-lasting analgesia and possible cognitive recovery benefits. However, further high-quality RCTs are required to confirm its long-term benefits and comparative efficacy within enhanced recovery protocols.

## PORT-SITE AND WOUND INFILTRATION

Port-site and wound infiltration is a simple, cost-effective technique widely used in laparoscopic surgeries. It involves injecting local anesthetics and, quite ironically, it is the only RA technique explicitly recommended by the PROSPECT guidelines (Grade D) [22]. A randomized controlled trial comparing port-site infiltration to the TAP block found no significant differences in pain scores, opioid consumption, or patient satisfaction, positioning port-site infiltration as a practical alternative due to its simplicity [46]. TAP combined with port-site infiltration added no benefit, supporting the efficacy of port-site infiltration alone [47]. It is important to highlight that the operating room team (both surgeon and anesthesiologist) must be fully informed about all techniques used, and must communicate this to each other. The type, volume, and concentration of local anesthetic used in wound/port side infiltration must always be shared with the anesthesia team, and in the same way, anesthesiologists should share the characteristics of the mixture in their own practice when applying perioperative RA techniques in order to avoid possible complications related to local anesthetic systemic toxicity (LAST).

Although comprehensive data in bariatric patients remain limited, at present, port-site infiltration is preferred over TAP blocks for its ease of application and effectiveness [22]. Further high-quality studies are needed to better define its role in enhanced recovery protocols for bariatric surgery and validate its long-term benefits in this high-risk population.

## SPECIAL CONSIDERATIONS—ULTRASOUND TECHNIQUE

The usage of fascial plane blocks has significantly improved perioperative pain management in bariatric surgery. The introduction of ultrasound guidance has not only enhanced the success rate of these blocks but also reduced the overall time needed to perform such procedures [48,49]. However, performing ultrasound RA in obese patients presents more technical challenges than in individuals not affected by obesity.

**FIGURE 1.**
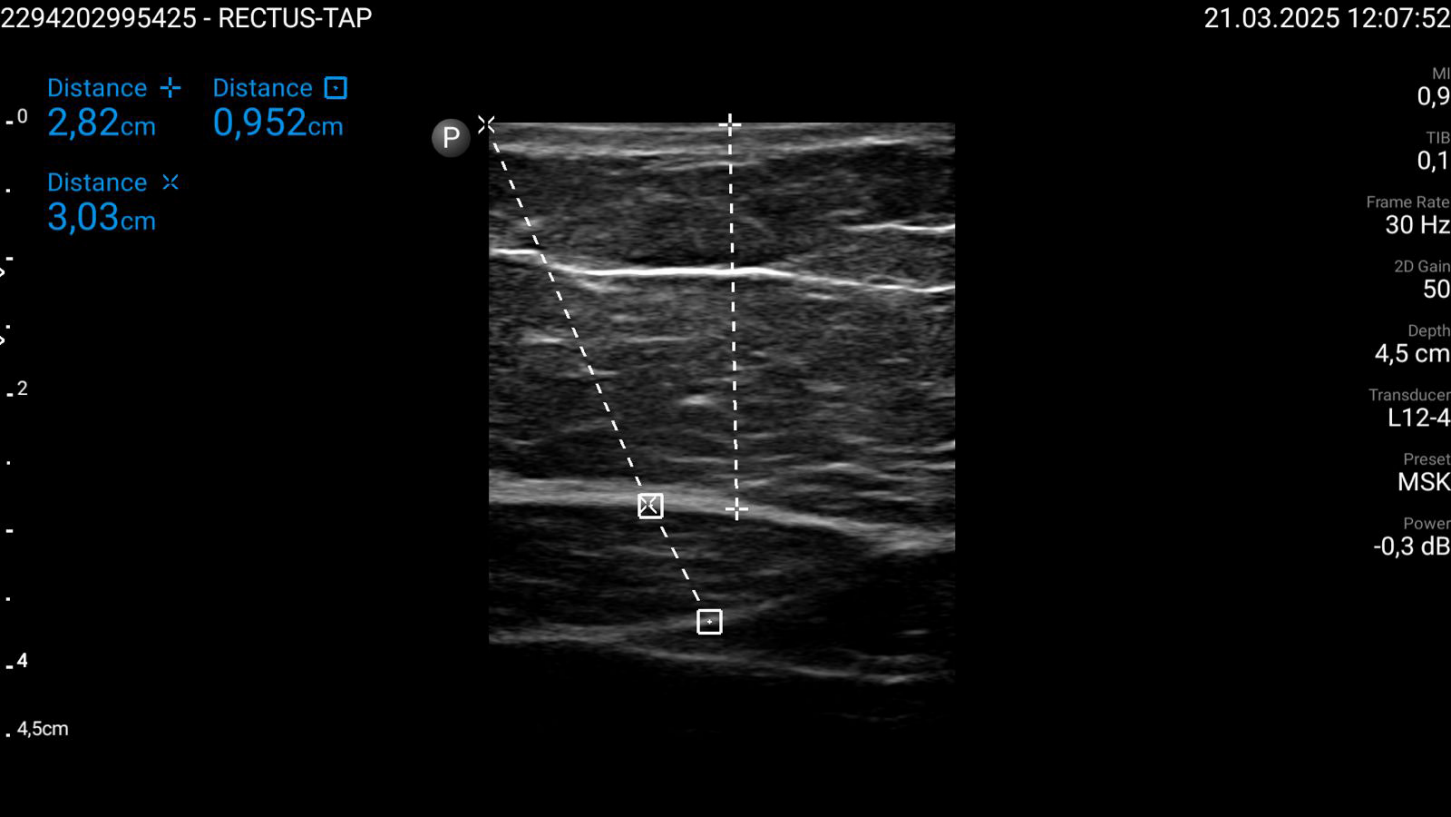
Ultrasound scan of the region of interest for performing a rectus sheath block in an obese patient. The ‘Cross’ represents the perpendicular distance from the skin to the rectus abdominis muscle, the ‘X’ indicates the hypothetical needle path from the skin to the rectus abdominis muscle, and the ‘Square’ denotes the rectus abdominis muscle.

**FIGURE 2.**
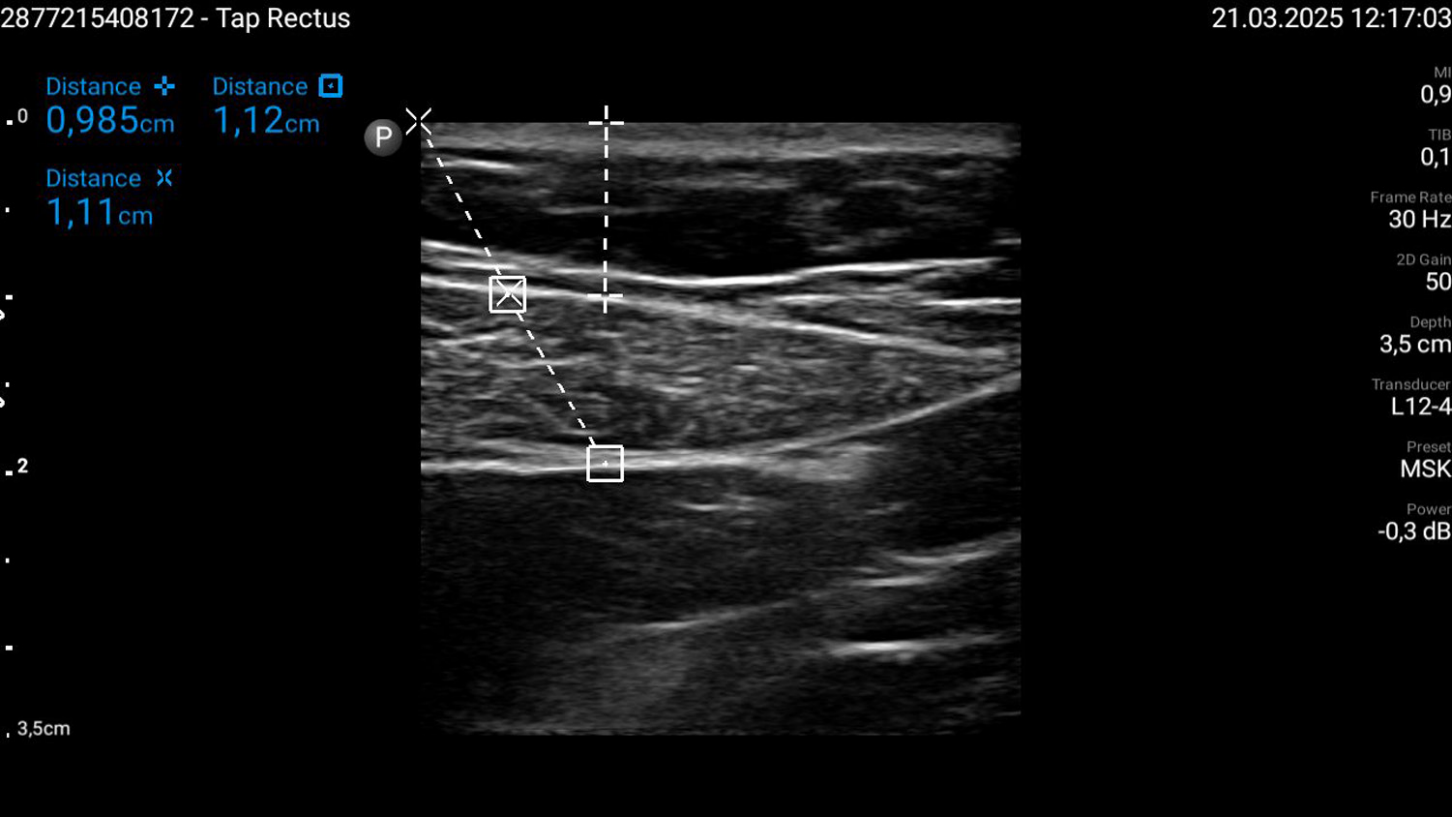
Ultrasound scan of the region of interest for performing a rectus sheath block in a normal weight patient obese patient. The ‘Cross’ represents the perpendicular distance from the skin to the rectus abdominis muscle, the ‘X’ indicates the hypothetical needle path from the skin to the rectus abdominis muscle, and the ‘Square’ denotes the rectus abdominis muscle.

The primary difficulty arises from the increased depth of target structures and the degradation of ultrasound image quality due to excess adipose tissue (Figs. 1 and 2). As the fat layer increases, ultrasound waves are attenuated by about 0.63 dB per centimeter of fat [50], reducing the visibility of anatomical landmarks and target structures, and the use of pads to enhance exposition of the area.

To optimize the use of ultrasound in obese patients, certain strategies can be employed. Increasing the frequency of the transducer may enhance resolution for superficial structures, while lower-frequency probes improve penetration for deeper targets. Adjusting the gain and depth settings can help compensate for beam attenuation caused by adipose tissue [48,49]. Techniques like the abdominal shift method or lateral decubitus positioning can improve visualization by reducing the distance between the probe and the target structure [51]. Furthermore, applying sufficient probe pressure may displace adipose tissue and enhance image clarity. These adjustments, combined with operator experience, can significantly improve the success rate of fascial plane blocks in this population [48,49]

## SPECIAL CONSIDERATIONS—LOCAL ANESTHETICS

In patients affected by obesity clinicians should calculate the appropriate dosage of local anesthetics for RA on LBW rather than total body weight (TBW) in order to reduce the risk of LAST. Obesity alters body composition, in fact fat mass contributes minimally to the distribution of local anesthetics, while LBW, which includes fat-free mass such as active cellular mass, extracellular water, and minerals, remains the primary compartment for drug distribution. Usually, in not-obese patients, 15–20% of TBW is fat weight, and 80–85% is LBW. However, in obesity, while TBW increases, LBW only accounts for 20–40% of the excess weight, and the fat mass increases disproportionately [52]. Additionally, it is difficult to provide a one fits all formula for all the local anesthetics as local anesthetics have different volumes of distribution depending on their lipophilic or hydrophilic nature, with lipophilic local anesthetics tending to distribute in fat mass and hydrophilic local anesthetics primarily in LBW [53]. Given the above, standard dosing methods based on TBW may lead to overdosage and increased risk of toxicity. Therefore, the maximum safe dose of LAs should be calculated based on LBW, especially for local anesthetics with a lower partition coefficient, such as chloroprocaine and mepivacaine [53]. Lastly, an additional factor of interindividual variability is due to altered pharmacokinetics in patients affected by obesity due to higher cardiac output, total blood volume, and changes in regional blood flow, which affect drug clearance and half-life. Thus, anesthesiologists should use the lowest effective dose, adjust doses based on LBW, and closely monitor for signs of toxicity or inadequate anesthesia [54].

## CONCLUSION

RA represents a cornerstone of multimodal analgesia strategies, especially for bariatric procedures. The integration of techniques such as TAP, QL, ESP blocks, and IPLA has shown promising results in reducing postoperative pain, opioid consumption, and complications. However, challenges related to anatomical variations in obese patients underline the necessity for skilled application and technological support, such as ultrasound guidance, to ensure the success and safety of these interventions. Future research should prioritize high-quality randomized controlled trials to further evaluate the comparative efficacy of different RA techniques in bariatric populations. Expanding the evidence base will enable more precise recommendations tailored to the unique needs of obese patients, fostering improved perioperative outcomes and aligning with enhanced recovery protocols.

## Acknowledgements

*None*.

## Financial support and sponsorship

*None*.

## Conflicts of interest

*None.*
